# How does the integration of cultural and tourism industries impact the value added to tourism value chain: Evidences from Jiangsu Province of China

**DOI:** 10.1371/journal.pone.0287610

**Published:** 2023-06-29

**Authors:** Meiling Zeng, Suyan Shen, Jie Gu

**Affiliations:** Department of Business Administration, College of Economics and Management, Nanjing Forestry University, Nanjing, Jiangsu Province, People’s Republic of China; Nanjing Audit University, CHINA

## Abstract

China has been fully implementing the policy of the cultural and tourism industrial integration since 2018. However, the value-added benefits of this policy are not prominent, and the relationship between industrial integration and the value added to the tourism value chain was seldom addressed by researchers. In the context of China’s high-quality development, it is necessary to conduct the impact of the integration of cultural and tourism industries on the value added to tourism value chain. This paper proposed four theoretical hypotheses and the corresponding econometric models based on the panel data from 2013 to 2020 in China’s Jiangsu Province. According to empirical results, the integration of cultural and tourism industries is spatially unbalanced, with notable imbalances between the south and the north. This paper identified a new connection between cultural and tourism integration and the tourism value chain. It is found that the integration of cultural and tourism industries can enhance the value added to tourism value chain either directly or indirectly through the information technology, with the direct effect being positively moderated by tourism agglomeration. Moreover, this paper may overturn how people generally think about the integration between cultural and tourism industries. It reveals a single-threshold effect that only when the integration of cultural and tourism industries reached a high level will it exert a positive effect. To be more specific, not all Chinese cities are suitable for implementing cultural and tourism integration, because the integration is likely to be ineffective in regions where the cultural industry is substantially less developed than the tourism industry.

## Introduction

Cultural tourism refers to tourism activities that involve heritage tourism [1] and creative tourism [2]. The value added of cultural tourism depends on the consumption activities occurred within the cultural scenic spots, which have generally a low value. The value added by cultural and tourism integration has been recognized in four parts [3]. Following the occurrence of industrial integration, actors in the tourism industrial chain will engage in new value-adding activities, resulting in the phenomenon of value reconstruction [4]. However, the existing studies of cultural and tourism integration rarely addressed this phenomenon, but mainly focused on the current situation and its action mechanism. Tourism value chain analysis has been regarded as a useful tool for assessing the impact of the tourism industry on developing countries [5]. It can help them identify the connections between tourism industry and the other industries [6]. Value chain governance and poverty reduction are two main topics addressed by the studies of tourism value chain, but there is a research gap on the relationship between cultural and tourism integration and the tourism value chain.

Nowadays, tourists tend to pursue more authentic experiences from tourism activities, so the boundaries between the cultural and tourism industries are gradually blurred and the scope of the tourism value chain is being expanded continuously [7]. The integration between cultural and tourism industries may promote other industries’ consumption due to the tourism multiplier effect [8], and realize industrial transformation and upgrading during the integration process. In some countries, cultural and tourism integration has been incorporated into the overall national economic development strategy [3]. China, for example, has not only released the policy of cultural and tourism integration but also implemented the corresponding institutional restructuring. Since then, China has been undergoing a boom in cultural and tourism integration all over the country. However, some problems have arisen during the integration process, such as the imbalance of industrial structure [9]. The use of value chain analysis can provide theoretical support for solving the problems caused by the integration between the two industries.

Taking Jiangsu Province of China as a case study, this paper aimed to explore the impact of the integration between cultural and tourism industries on the value added to the tourism value chain. Specifically, it addressed the following issues: (a) what is the integration level of cultural and tourism industries. (b) whether this integration level impacts tourism value chain and whether there is any heterogeneity; and (c) how does the industrial integration impact tourism value chain adding. From the perspective of tourism value chain, this paper provided creative responses to the question as how to effectively implement cultural and tourism integration. First, on the basis of the value chain theory, a new connection between cultural and tourism integration and the tourism value chain was identified, which was in line with China’s national strategies of high-quality development. Second, the general impression that cultural and tourism integration can bring over significant benefits was overturned. It was revealed that only high-level integration between the cultural and tourism industries might lead to value addition.

The structure of this paper is as follows: Section 2 provides literature reviews on the related topics; Section 3 proposes four theoretical hypotheses; Section 4, Section 5 and Section 6 present the empirical process and results; and Section 7 is the conclusions and implications.

## Literature review

### Integration of cultural and tourism industries

The integration between cultural and tourism industries is a common market phenomenon, but not much research has been conducted on this topic yet. The existing literature mainly focuses on two aspects: the evaluation of the current situation and the action mechanism in different aspects.

On the one hand, researchers have evaluated the current situation of the regional integration of cultural and tourism industries by measuring the spatial and temporal evolution. Grey relational analysis, data mining [10], and the coupling coordination degree model [9, 11, 12] are common models that can reflect the differences of regional development of cultural and tourism industries. For example, it was reported that the cultural industry in Guangxi province [12] was developing slightly faster than the tourism industry, but Shaanxi [9] lagged behind the tourism industry.

On the other hand, driving factors and influence paths have emerged as hotspots in the research of the related field. The integration between cultural and tourism industries has apparent advantages in promoting industrial and economic development [13]. With the development of digital economy, information technology has rebuilt the tourism value chain [14] and greatly improved the tourism efficiency [15]. According to the available literature, researchers have not comprehensively discussed the necessity of the integration between cultural and tourism industries, nor have they elaborated how to do it from the perspective of high-quality tourism development.

### Tourism value chain

Value chain analysis is widely used in the research of manufacturing industry based on Porter’s value chain theory. However, the uniqueness of the tourism industry makes it necessary to specially define tourism value chain rather than directly adopting the value chain theory of the manufacturing industry. According to Hjalager [16], there are supply-oriented and demand-oriented definitions for tourism value chain. From the supply-oriented perspective, tourism value chain can be considered as the supply chain of tourism products based on Porter’s value chain model. It involves four types of stakeholders [17]. From the demand-oriented perspective, tourism value chain has the meaning of Value Shop [18] and is a continuum of the related economic activities associated with visitors [19].

Tourism value chain involves multiple actors and the distribution of value among multiple industries. Through value chain analysis, the dynamic flow of economic and organizational activities among actors of different industries can be easily uncovered by focusing on the inter-linkage. Most of the studies on tourism value chain are qualitative analyses of the governance model [5, 17], the rural tourism value chain reconstruction [20], and the value co-creation model [21]. In addition, there are also a few scholars who quantitatively analyzed the impact of tourism value chain on tourism poverty alleviation [22], regional economic leakage [23], and tourism destination management [18]. However, how to improve the regional tourism value chain from the perspective of industrial integration was rarely addressed. In fact, this is the perspective that can best reflect the concept of value chain.

## Theoretical hypotheses

### The relationship between industrial integration and tourism value chain

The integration of cultural and tourism industries refers to the process of mutual penetration, continuous reorganization, and optimization of culture and tourism elements [3], which can promote tourism value chain from three aspects, i.e., integration of markets, integration of resources, and integration of supply chains.

Firstly, the market of cultural industry has been highly valued by the suppliers due to its excellent quality, high consumption potential, and generally-steady visitor flow [15]. Markets integration can promote the transfer of tourism value chain to high-consumer groups and improve consumption level. The integration of cultural and tourism industries can also optimize the entire value chain by encouraging the participation of tourists from design, manufacturing, management, marketing, and other multi-links [24]. Secondly, the advantage of cultural and tourism integration lies in the combination of the aesthetic value of cultural resources with the experience value of tourism resources. Such integration can facilitate the flow of resources and value restructuring, which means that materials, knowledge, and human resources will be allocated to higher-value parties for the purpose of generating higher returns [25]. Finally, the primary tourism-related industries, covering the six elements of tourism (i.e., food, accommodation, transportation, sightseeing, shopping, and entertainment), used to be the essential participants and the main value-added body in the tourism value chain. But now, they have declined to the low end of the value chain because of low barriers to entry, limited resources renewal, and insufficient knowledge. The integration of supply chains between cultural and tourism industries implies that resources and values are redistributed among different actors. It can help the primary tourism-related industries evolve to a higher value-added stage in the value chain and, at the same time, add value to the whole tourism value chain.

**Hypothesis 1a.** The integration of cultural and tourism industries can promote the value added to tourism value chain.

Tourism has evolved into an important stage featuring the transformation from high-speed to high-quality development [26]. However, not every area’s tourism has advanced to the point of high-quality development. One key element determining the high-quality growth of tourism is the degree of industrial integration. High-level integration of cultural and tourism industries is a comprehensive concept covering cultural tourism resources, facilities and environments [27]. By supplying effectively, it can meet market demands and guide consumptions. Contrarily, low-level integration of cultural and tourism industries describes the early stages of integration that are low-coupling, uncoordinated, and characterized by disparities between supply and demand [28]. Over-commercialization and homogeneity might become issues as a result. The resource dependency theory suggests that enterprises with superior resources have the power to determine the flow of resources in competing relationships. Therefore, the uncoordinated development of cultural industry and tourism industry is not conducive to the efficient and reasonable flow of information, resources and other factors.

**Hypothesis 1b.** The value-added effect of high-level integration between cultural and tourism industries is superior to that of low-level integration.

### The moderating effect of tourism agglomeration

Tourism agglomeration refers to the spatial proximity of tourism businesses on the basis of the relationship in the supply chain, which emphasizes the importance of value chain and spatial agglomeration [29]. In view that the quantity and variety of products in a tourism destination have an obvious effect on tourists’ choices [30], stronger tourism agglomeration means a more variety of goods to serve tourists and a higher value of experience gained by tourists. In addition, tourism agglomeration can be utilized as a collaboration platform among local tourism companies [31]. It can accelerate the flow of various factors in the agglomeration space to form the scaling effect through the mechanisms of information sharing, resource allocation, talent exchange and policy support across enterprises [12]. The higher the degree of tourism agglomeration, the greater the value added of tourism value chain is. Therefore, tourism agglomeration adds value not only to the various products clustered by enterprises, but also to the tourists’ experience.

**Hypothesis 2.** Tourism agglomeration can intensify the promoting effect of integration between cultural and tourism industries on the value added to tourism value chain and play a positive moderating role.

### The mediating effect of information technology

Information technology aids industrial integration, which will tear down barriers across industries. The integration of cultural and tourism industries has been substantially improved by information technology, especially during the epidemic when tourist activities are restricted [13]. It can play positive effect by achieving product innovation and precise marketing. Firstly, information technology plays a significant role in knowledge creation [32]. Technologies such as VR and AR are carriers of invisible cultural products, which are helpful for the innovation of tourism products by breaking through the temporal and spatial restrictions. Thus, tourists can experience the dialogue with history and activate the traditional culture. Secondly, tourism is a very information-intensive activity [33]. Information technology not only affects tourists’ information acquisition and consumption decision making, but also plays a positive role in the marketing of suppliers [34]. Due to the intangibility and unpredictability of tourism products, tourists need to collect adequate information when choosing their desired products. At the same time, tourists’ demands are also diverse and volatile, so the supplies of tourism products and services are required to respond to market changes in a timely manner. The market orientation of actors guiding the production process at multiple stages of the chain based on market information is the prerequisite for the creation of value [35].

**Hypothesis 3.** The integration of cultural and tourism industries achieves value added to tourism value chain through information technology. It plays a mediating role in this process.

## Methods

### The coupling coordination degree evaluation model

Coupling refers to the motion of a system where the subsystems interact with each other, and coordination means the relationship between subsystems that work together in a harmonious way [36]. The coupling coordination degree (CCD) has been widely used to measure the level of industry integration. Although the conventional concept of CCD has been studied by plenty of scholars, it involves two obvious problems [37]. Firstly, the coupling model didn’t satisfy the assumption of normal distribution but was explained by it. Secondly, the contribution coefficients of the coordination model were defined in an artificial and subjective way, which simplified the model but deviated from the true level. Correspondingly, Wang [37] modified the conventional coupling model and solved the first problem, while Shen [38] proposed an improved coordination model to address the second problem. Therefore, the improved CCD models were used to measure the integration between cultural and tourism industries. The detailed calculation steps are as follows.

First, the information entropy method is applied to calculate the weights of indicators. It assigns a weight to each indicator mainly on the basis of the information contained in this indicator rather than the data linearity [39], which can avoid bias by subjective influence.

Standardize each positive indicator to eliminate the differences in units.


$$M_{ij}=\frac{X_{ij}-m{in}_{j}(X_{ij})}{\max_{j}\left(X_{ij}\right)-m{in}_{j}(X_{ij})}+0.001$$ (1)

Calculate the proportion of the indicator j in the sample i.


$$P_{ij}=\frac{M_{ij}}{\Sigma_{i=1}^{m}M_{ij}}$$ (2)

Calculate the information entropy of the indicator j.


$$e_{j}=-\frac{1}{\ln\left(m\right)}\sum_{1}^{m}P_{ij}\ln\left(P_{ij}\right)$$ (3)

Calculate the weight of the indicator j.


$$w_{j}=\frac{{1-e}_{j}}{\Sigma_{i=1}^{n}{1-e}_{j}}$$ (4)

Where *M*_*ij*_ and *X*_*ij*_ denote the standardized value and the original value of indicator j in the sample i, respectively; *max*_*j*_(*X*_*ij*_) and *min*_*j*_(*X*_*ij*_) refer to the maximum and minimum value of indicator j among all the samples, respectively; n refers to the number of indicators in each system; m refers to the total sample covering all the cities over the entire observation period.

Second, the technique for order preference by similarity to an ideal solution (TOPSIS) can reflect the relative importance of each indicator with the time sequence. It serves an effective tool to evaluate the development degree of a subsystem [36].

Calculate the distance from a sample to the positive ideal solution and negative ideal solution.


$$\left\{ \begin{array}{l} d_{j}^{+}=\sqrt{\sum_{j=1}^{n}w_{j}{\left(M_{ij}-M_{j}^{+}\right)}^{2}}\\ d_{j}^{-}=\sqrt{\sum_{j=1}^{n}w_{j}{\left(M_{ij}-M_{j}^{-}\right)}^{2}} \end{array}\right.$$ (5)

Where $M_{j}^{+}$ and $M_{j}^{-}$ refer to the maximum and minimum value of indicator j among all the samples, respectively.

Calculate the relative closeness of a sample to the ideal solution.


$$c_{i}=\frac{d_{i}^{-}}{d_{i}^{+}+d_{i}^{-}}$$ (6)

Finally, the improved CCD is used to reflect the integration of the two industries.

Calculate the coupling degree following Wang’s [37] modification.

Assuming max *U*_*i*_ is *U*_*c*_, then

$$C=\sqrt{1-(U_{c}-U_{t})\times \frac{U_{t}}{U_{c}}}$$ (7)

Where *U*_*c*_ and *U*_*t*_ refer to the comprehensive development level of the cultural industry and tourism industry measured by the relative closeness *c*_*i*_, respectively.

Calculate the coordination degree following Shen’s [38] modification.


$$\left\{ \begin{array}{c} T=\alpha \times U_{c}+\beta \times U_{t}\\ \alpha =\frac{U_{t}}{U_{C}+U_{t}}\\ \beta =\frac{U_{C}}{U_{C}+U_{t}} \end{array}\right.$$ (8)

Where *α* and β are contribution coefficients of the two systems, meeting the condition of α+β = 1.

Calculate the improved CCD as follow.


$$D=\sqrt{C\times T}$$ (9)

### Index selection

The evaluation index system was used to calculate the CCD of the cultural and tourism industries in this paper. According to the industrial integration mechanism, the integration of cultural and tourism industries was evaluated in terms of markets integration, supply chains integration and resources integration. Considering the consistency and availability, 14 indicators were selected from 5 aspects: total market revenue, reception volume, core industry income, number of core industries, and number of core resources (see Table 1). The results are shown in S1 Table.

**Table 1:** Evaluation index system for the cultural industry and tourism industry.

Cultural industry	Markets integration	Total revenue of cultural market
		Visitors of cultural activities
	Supply chains integration	Operating income in cultural-related industries
		Employees in cultural-related industries
	Resources integration	Number of libraries
		Number of museums
		Number of cultural centers
Tourism industry	Markets integration	Total tourism revenue
		Number of domestic and foreign tourists
	Supply chains integration	Sum of operating income in travel agencies, star hotels and A-grade tourist attractions
		Sum of employees in travel agencies, star hotels and A-grade tourist attractions
	Resource integration	Number of travel agencies
		Number of star hotels
		Number of A-grade tourist attractions

## Research design

### Study area

Located in the eastern part of China, Jiangsu is a major economic province nourished by the Yangtze River and the Grand Canal. It is home to several UNESCO world heritage sites, including China’s Grand Canal, Suzhou classical gardens, and Ming Xiaoling Mausoleum, as well as intangible cultural heritages like Kunqu Opera, woodblock and Yunjin embroidery. With a long history, profound culture and picturesque natural sceneries, each city in Jiangsu has its distinctive characters, making Jiangsu a huge tourism market in China. In 2019 (before Covid-19), Jiangsu received 3.9 million overseas tourists and earned $4.74 billion in foreign exchange earnings from tourism. During the May Day holiday in 2023 (the first major public holiday in China after the pandemic), Jiangsu received 39.8 million tourists and brought in tourism revenue of 9.96 billion yuan, ranking the first over the country. Besides, the “Charm of Jiangsu” brand has spread out of China as one of the top three most influential international tourism brands. As one of the most popular tourism destinations, Jiangsu has made outstanding achievements in cultural tourism festivals, performing arts programs and utilization of intangible cultural heritages.

### Model specification

The data used in this paper covers 13 cities of Jiangsu Province over a period of 8 years. A fixed effect regression model with a controlling year effect was employed for analysis. Because heteroskedasticity, autocorrelation and cross-section relation might cause biased estimates, the regression with Driscoll-Kraay standard errors was used in the fixed effect model to obtain valid and consistent unbiased estimates as far as possible.

First, the main regression model established for the impact of integration between cultural and tourism industries on the value added to tourism value chain is as follows.


$${VALUE}_{it}=a_{0}+\beta_{1}{CCD}_{it}+\beta_{2}{TA}_{it}+\lambda_{k}{Controls}_{it}+u_{i}+t_{i}+\varepsilon_{i}$$ (10)

Where i denotes the city and t denotes the year; *a*_0_ is a constant, and *β*_1_, *β*_2_, *λ*_*k*_ are regression parameters to be estimated; *u*_*i*_ refers to the unobserved individual effect; *t*_*i*_ refers to the year effect; *ε*_*i*_ is the random error term.

Second, the panel threshold model is used to analyze the heterogeneity impact of the integration of cultural and tourism industries on tourism value chain. According to the statistical effect, this model may categorize industrial integration into low and high phases by describing the leaping character or structural break in the link between different variables [40]. The panel threshold regression model is established as follows.


$${VALUE}_{it}=a_{0}+\gamma_{1}{CCD}_{it}\cdot I({CCD}_{it}\leq \delta )+\gamma_{2}{CCD}_{it}\cdot I({CCD}_{it}\geq \delta )+\beta_{2}{TA}_{it}+\lambda_{k}{Controls}_{it}+u_{i}+t_{i}+\varepsilon_{i}$$ (11) s

Third, to test the moderating effect of tourism agglomeration on the value added of industrial integration to tourism value chain, an interaction term (CCD*TA) is introduced. The moderation analysis model is established as follows.


$${VALUE}_{it}=a_{0}+\beta_{1}{CCD}_{it}+\beta_{2}{TA}_{it}+\beta_{3}{CCD*TA}_{it}+\lambda_{k}{Controls}_{it}+u_{i}+t_{i}+\varepsilon_{i}$$ (12)

The mediating effect is an intermediate path underlying the effect of X to Y. Specifically, X influences the mediator variable M (path a is described by Eq (13)), which in turn influences Y (path b is described by Eq (14)). If the coefficients of *a*_1_ and *b*_2_ are statistically significant, the mediating effect can be established.


$${IT}_{it}=i_{1}+a_{1}{CCD}_{it}+a_{2}{TA}_{it}+a_{k}{Controls}_{it}+u_{i}+t_{i}+\varepsilon_{i}$$ (13)

$${VALUE}_{it}=i_{0}+b_{1}{CCD}_{it}+b_{2}{IT}_{it}+b_{3}{TA}_{it}+\lambda_{k}{Controls}_{it}+u_{i}+t_{i}+\varepsilon_{i}$$ (14)

Finally, Instrumental Variable Estimates are widely used in solving almost all types of endogenous problems. In this paper, the two-stage least-squares (2SLS) estimates were employed for robust test.

### Variable measurement

The integration level of cultural and tourism industries was taken as the main explanatory variable in this paper, which is calculated through the improved CCD as above. The CCD can reflect the process of benign interaction and synergistic development of the two industries.

The value added to tourism value chain was selected as an explained variable. Hjalager [16] proposed to measure the value chain as the difference between turnover and costs, i.e., profit. Combining the concept of tourism value chain and the data from statistical yearbooks, the total tourism revenue can be considered as the total value paid by tourists, i.e., the turnover of the tourism industry. The operating costs of the tourism industry can be reflected by the GDP contributed by the six elements of tourism (i.e., food, accommodation, transportation, sightseeing, shopping, and entertainment) based on the expenditure method. The subtraction of the two derives the final value addition to tourism value chain. Therefore, the value added to tourism value chain can be calculated as follows.


$$TVC=TTR-{GDP}_{i}$$ (15)

Where TVC refers to the value added to tourism value chain. TTR refers to the total tourism revenue. *GDP*_*i*_ refers to the GDP of industry i which belongs to one of the six elements of tourism. The *Chinese System of National Account* divides industries and measures GDP based on the production at the supply side, while the six elements of tourism are defined based on the tourists’ demand. Such a mismatch shows that tourism value chain is actually concealed in different economic departments [31]. For example, the transportation industry is involved for providing tourist transportation services; the accommodation and catering industry is involved for providing tourist catering and accommodation services; the retail industry is involved for providing tourist shopping services; and the entertainment and recreation industry is involved for providing tourist sightseeing and entertainment services. Therefore, Eq (15) can effectively reflect the value added to tourism value chain.

As a moderated variable, tourism agglomeration reflects the scaling effect of tourism industry and the intensity of tourism activities. Thus, the total tourism revenue divided by regional GDP was used in this paper to measure tourism agglomeration [41, 42]

Information technology is a mediating variable. From the industrial level, the postal business volume [29] has difficulty in reflecting the development level of 5G Internet. The Internet penetration rate [14] ignores the overall digital economy. Therefore, this paper used the ratio of the GDP of the information transmission, computer services and software industry to the total regional GDP as the proxy variable for information technology. A larger ratio refers to a higher development level of regional informatization and greater opportunities to apply information technology in other industries.

The traffic passenger volume, the upgrading of industrial structure, and the government consumption were selected as control variables. The traffic passenger volume reflects the tourism traffic accessibility [43], which needs to be guaranteed by transportation infrastructure. The upgrading of industrial structure was measured by the industrial structure supererogation. An upgraded industrial structure can play a positive role in improving the efficiency of the tourism industry [44]. Government consumption reflects the government support for economic development, which is measured by the ratio of fiscal expenditure to regional GDP as a proxy variable [14].

### Data source and descriptive statistics

The data used in this paper is the panel data collected from 13 cities of Jiangsu province from 2013 to 2020 (Jiangsu began to conduct statistics on culture-related industries since 2013). As macroscopic statistical data is characterized by authenticity, objectivity and comparability, it is suitable for horizontal and vertical analyses. Thus, all the data used in this paper was directly collected or calculated from *Jiangsu Statistics Yearbook*, *Jiangsu Culture and Tourism Statistics Yearbook*, *China Statistics Yearbook*, and the *Statistics Yearbooks* of the 13 cities. The raw data used for analysis of this paper is as shown in S2 Table. The definitions and descriptive statistics on the variables used in this paper are shown in Table 2.

**Table 2:** Definitions and descriptive statistics of the variables.

Variable	Measurement	Obs	Mean	Std.Dev.	Min	Max
Explained Variable						
VALUE	Total tourism revenue subtracts the GDP of the six elements of tourism	104	171.4	358.5	-445.5	1297
Explanatory Variable						
CCD	The CCD of cultural industry and tourism industry	104	0.363	0.158	0.176	0.856
Moderating Variable						
TA	Total tourism revenue divided by regional land area	104	0.299	0.256	0.0341	1.248
Mediating Variable						
IT	GDP of the information technology industry divided by regional GDP	104	0.022	0.016	0.011	0.093
Control Variable						
TRANS	Logarithm of traffic volume	104	9.043	0.653	7.640	10.810
GOV	Fiscal expenditure divided by regional GDP	104	0.124	0.0293	0.0851	0.200
ISS	GDP of the tertiary industry divided by the GDP of the secondary industry	104	1.037	0.190	0.763	1.785

## Empirical findings and discussions

### The CCD of cultural and tourism industries

Table 3 presents the CCD of cultural and tourism industries in 13 cities of Jiangsu province. According to Geng [36], the CCD can be classified into 8 categories. By observing the average value of the 13 cities (see S1 Fig), it was found that the coupling level of Jiangsu was basically balanced, but the coordination level was imbalanced. Only 3 cities achieved a balanced development between the two industries. Consequently, the integration between the cultural and tourism industries was still of a low quality in Jiangsu province. In particular, the development of the cultural industry generally lagged behind the tourism industry, which seriously restricted the progression towards high-level integration [9]. The low-level integration between the two industries is not conducive to the value added to the whole tourism value chain.

**Table 3:** The status of the integration between culture and tourism industries in Jiangsu province.

City	CCD	Coupling	Coordination	Cultural industry	Tourism industry	Integration level
Nanjing	0.738	0.834	0.656	0.640	0.691	Intermediately coordinated
Wuxi	0.533	0.777	0.368	0.312	0.451	Reluctantly coordinated
Suzhou	0.521	0.596	0.456	0.348	0.666	Reluctantly coordinated
Changzhou	0.435	0.824	0.231	0.201	0.274	Approaching imbalanced
Xuzhou	0.370	0.741	0.185	0.153	0.245	Slightly imbalanced
Nantong	0.367	0.777	0.174	0.147	0.218	Slightly imbalanced
Yancheng	0.303	0.886	0.104	0.094	0.117	Slightly imbalanced
Huaian	0.293	0.890	0.097	0.089	0.108	Moderately imbalanced
Taizhou	0.283	0.922	0.087	0.086	0.090	Moderately imbalanced
Suqian	0.249	0.878	0.072	0.083	0.066	Moderately imbalanced
Yangzhou	0.211	0.462	0.097	0.062	0.236	Moderately imbalanced
Zhenjiang	0.207	0.477	0.090	0.057	0.216	Moderately imbalanced
Lianyungang	0.205	0.610	0.070	0.049	0.123	Moderately imbalanced
Total	0.363	0.744	0.207	0.179	0.269	Slightly imbalanced

Fan and Xue [9] found that the integration of cultural and tourism industries in Shaanxi had high and low-value clusters. Similarly, the integration of cultural and tourism industries in Jiangsu also showed a clear spatial difference (see S2 Fig), which is mainly attributed to the factors of resource endowment and industrial foundation [45]. The cities in Jiangsu can be divided into three tiers in terms of integration level. The first tier includes Nanjing, Wuxi and Suzhou, whose cultural and tourism industries have developed in good coordination due to their strong economy and profound historical culture. As a result, the integration between cultural and tourism industries has effectively promoted their industrial value addition and generated positive spillover effects [12]. The second tier includes Changzhou, Xuzhou, Nantong and Yancheng, whose industrial integration is slightly imbalanced. In general, the development of their cultural industry lags slightly behind that of the tourism industry, but the potential of industrial integration is enormous. The third tier includes Huaian, Taizhou, Suqian, Yangzhou, Zhenjiang and Lianyungang, whose industrial integration is moderately imbalanced. In these cities, both the cultural and tourism industries are at a low development level, lacking of driving force from advantageous industries. Yangzhou and Zhenjiang are two exceptions in the third-tier cities, as the development of their tourism industry is at a much higher level than that of their cultural industry. However, the significant development gap between the two industries greatly weakens the value added to the tourism value chain. The basic goal of high-quality development is to achieve fair and mutual benefits [46]. The cultural and tourism industries must achieve a coordinated development for a high-level integration and high value addition.

### The impact of industrial integration on tourism value chain

According to Table 4 Model 1 illustrates the relationship between the cultural and tourism integration and the value added to tourism value chain. It can be seen that the integration between cultural and tourism industries has contributed to the value added to tourism value chain at the 1% statistic level, with the regression coefficient of 1,252.262. In terms of the economic significance, every increase by one unit of the level of CCD will improve the value added to tourism value chain by 1,252.262 units. Therefore, hypothesis 1a was supported.

**Table 4:** Regression results of the impact of cultural and tourism integration on the tourism value chain and its mechanism.

MODEL	(1)	(2)	(3)	(4)	(5)	(6)
	FE	TR	MOD	MED1	MED2	IV
VARIABLES	VALUE			IT	VALUE	
CCD	1,252.262***		1,126.785**			1,098.118**
	(4.60)		(3.12)			(2.23)
CCD(CCD<0.4657)		-447.891				
		(-0.92)				
CCD(CCD>0.4657)		2499.591***				
		(5.88)				
DUM_CCD				0.010***	176.446***	
				(3.28)	(2.71)	
TA	1,051.395***	1,008.746***	847.605***	-0.008*	716.235***	1,037.821***
	(15.94)	(7.41)	(43.50)	(-1.76)	(7.90)	(6.64)
CCD*TA			2,768.672***			
			(5.27)			
IT	-1,373.425	-9,499.273***	-3,788.082***		9,885.303***	-975.412
	(-0.93)	(-3.55)	(-4.85)		(4.78)	(-0.39)
TRANS	5.030	-29.467	-37.734	-0.003*	-34.431	7.245
	(0.13)	(-0.72)	(-1.49)	(-1.77)	(-0.97)	(0.16)
GOV	226.967	-565.890	101.489	-0.063*	1,884.945***	371.272
	(0.17)	(-0.57)	(0.11)	(-1.83)	(2.71)	(0.32)
ISS	21.508	-105.704	-58.835	0.070***	244.451	49.324
	(0.18)	(-0.51)	(-0.80)	(12.28)	(1.33)	(0.21)
CONSTANT	-645.358	231.968	-56.073	-0.007	-425.578	
	(-1.37)	(0.46)	(-0.17)	(-0.38)	(-1.20)	
R-squared	0.814	0.857	0.866	0.787	0.841	0.814
Year control	YES	YES	YES	YES	YES	YES
First stage of F						118.51***
F	18509.54***	33.02***	8437609.86***	28.01***	36.55***	25.81***

t-statistics in parentheses *** p<0.01

** p<0.05

* p<0.1

Under global value chain governance, there are four approaches to increase the industry value: process upgrading, product upgrading, internal chain upgrading, and inter-industry upgrading [35, 47]. The integration between cultural and tourism industries is the embodiment of cross-industry restructuring, which can add value to the tourism value chain in three ways. Firstly, a high-consumption cultural tourism market has been developed, which has greatly increased the income from tourism-related industry. Secondly, the integration of cultural and tourism resources has added value by embedding diverse experience values and increasing the efficiency of resource allocation. And thirdly, the integration of cultural and tourism supply chains has enhanced the value added via the upgrading of industrial structures.

Of course, the integration between cultural and tourism industries also has negative effects on the tourism value chain. Firstly, the culture and tourism integration advocates resources sharing between hosts and guests, which may exhaust local public resources for the local residents, increase their costs of living and reduce their quality of life [48]. Secondly, the integration may cause a surge in market entities in each link of the tourism value chain, and the homogeneity in cultural resources can easily lead to vicious competition, such as excessive commercialization. Thirdly, while pursuing economic benefits, the protection and inheritance of local culture may be neglected [49]. Commercialization of cultural resources through tourism development can easily distort the local culture, leading to the disappearance of cultural authenticity [50, 51].

### The heterogeneity impact of industrial integration on tourism value chain

As indicated in Table 4 the integration of cultural and tourism industries may be divided into two phases depending on the selected threshold values from Model 2. It can illustrate the heterogeneous influence of industrial integration on the impact of value added to tourism value chain.

From Table 5 the estimator of the single-threshold model is 0.467 (P = 0.022), while that of the double-threshold model fails to reject the null hypothesis (P = 0.440). It indicates that the integration of cultural and tourism industries has a single-threshold effect. Model 2 demonstrates that high-level integration of cultural and tourism industries (higher than 0.466) has a positive effect on the value added to tourism value chain, which has passed the significance of 1%-level test. Surprisingly, the low-level integration of cultural and tourism industries (lower than 0.466) may reduce the value added to tourism value chain although failing the statistical test. The results supported hypothesis 1b.

**Table 5:** The results of panel threshold regression.

	Threshold	F	confidence interval
Single threshold	0.466	28.850**	[0.463,0.467]
Double threshold	0.567	17.320	[0.552,0.574]

The findings above suggest that the negative effect of the integration of cultural and tourism industries tends to predominate when the CCD of cultural and tourism industries is lower than 0.466. This threshold value is very close to that of Shi *et al*. [27] for high-quality cultural tourism in the Yangtze River Delta (0.46). Meanwhile, the integration of cultural and tourism industries may differ from other industries’ integration. For example, the integration between the AI and energy industries only needs to be coupled rather than coordinated to achieve a 20% increase in annual growth rate for the energy industry [52]. In comparison, the integration between cultural and tourism industries has to be both coupled and coordinated to achieve sustainable development [53]. Nevertheless, the negative impact does not deny the rationality of the China’s policy of culture and tourism integration but rather confirms the need for in-depth researches. Accordingly, future researches should shift from the argument on whether to implement the policy of culture and tourism integration to how to promote the cultural and tourism industries toward high-level integration.

### The moderating effect of tourism agglomeration

Model 3 (Table 4) with an additional interaction term coefficient (CCD*TA) on the basis of Model 1, estimates the moderating effect of tourism agglomeration, and centralized treatment was performed to avoid multicollinearity. The results of Model 3 indicate that the interaction term coefficient (CCD*TA) is 2768.672, with the P-value suggesting statistically significant. Meanwhile, the variables of TA and CCD have both passed the significance test and show the same direction, implying that tourism agglomeration plays an assisting role rather than a substituting role. Therefore, hypothesis 2 was supported.

The moderating effect of tourism agglomeration suggests that tourism agglomeration can promote the cultural and tourism industries to the high-level integration. This is consistent with the conclusion of Yan *et al*. [54], which argued that the scaling effect of the logistics industry would enhance the coupling quality of the two industries. The industrial agglomeration [55] and agricultural agglomeration [56] have posed both a positive spillover effect and negative crowding effect on economic sustainability. Compared to other industries, the negative crowding impact of tourism agglomeration is not obvious. This is because the value added of the tourism value chain is contributed from the tourists. The clustering of tourism-related companies can help enrich the types of tourism supply and reduce the average transportation cost [57]. Furthermore, tourism agglomeration stems from the attractiveness of tourism destinations, on the basis of strong attraction to enterprises and tourists [57]. The tourism flow formed by the gathering of tourists has an external spillover effect, which can promote the integration of culture and tourism industries across multiple regions.

### The mediating effect of information technology

The causal mediation analysis proposed by Imai *et al*. [58] accords with causal inference and is suitable for testing the causal mediation mechanism. Thereby, this paper applied the dummy variable of CCD (1, if higher than the threshold value; otherwise, 0) for exposure to treatment, and performed sensitivity analysis as robust test. As shown in Model 4 and Model 5 from Table 4 the test of CCD to IT (path a), and the joint test of CCD and IT to VALUE (path b) are both statistically significant. As shown in Table 6, the estimated coefficient of ACME is 106.334, with the 95% confidence interval [45.059,178.377] failing to include 0, implying that the integration of cultural and tourism industries partially promotes the value added to tourism value chain via information technology at the level of 36.611%. Therefore, hypothesis 3 was supported.

**Table 6:** The results of information technology based on causal mediation analysis.

	Mean	[95% Conf. Interval]
ACME	106.334	[45.059, 178.377]
Direct effect	175.652	[72.601, 312.298]
Total effect	281.985	[157.811, 401.878]
Mediated proportion	36.611%	[0.265, 0.674]

**Fig 1:**
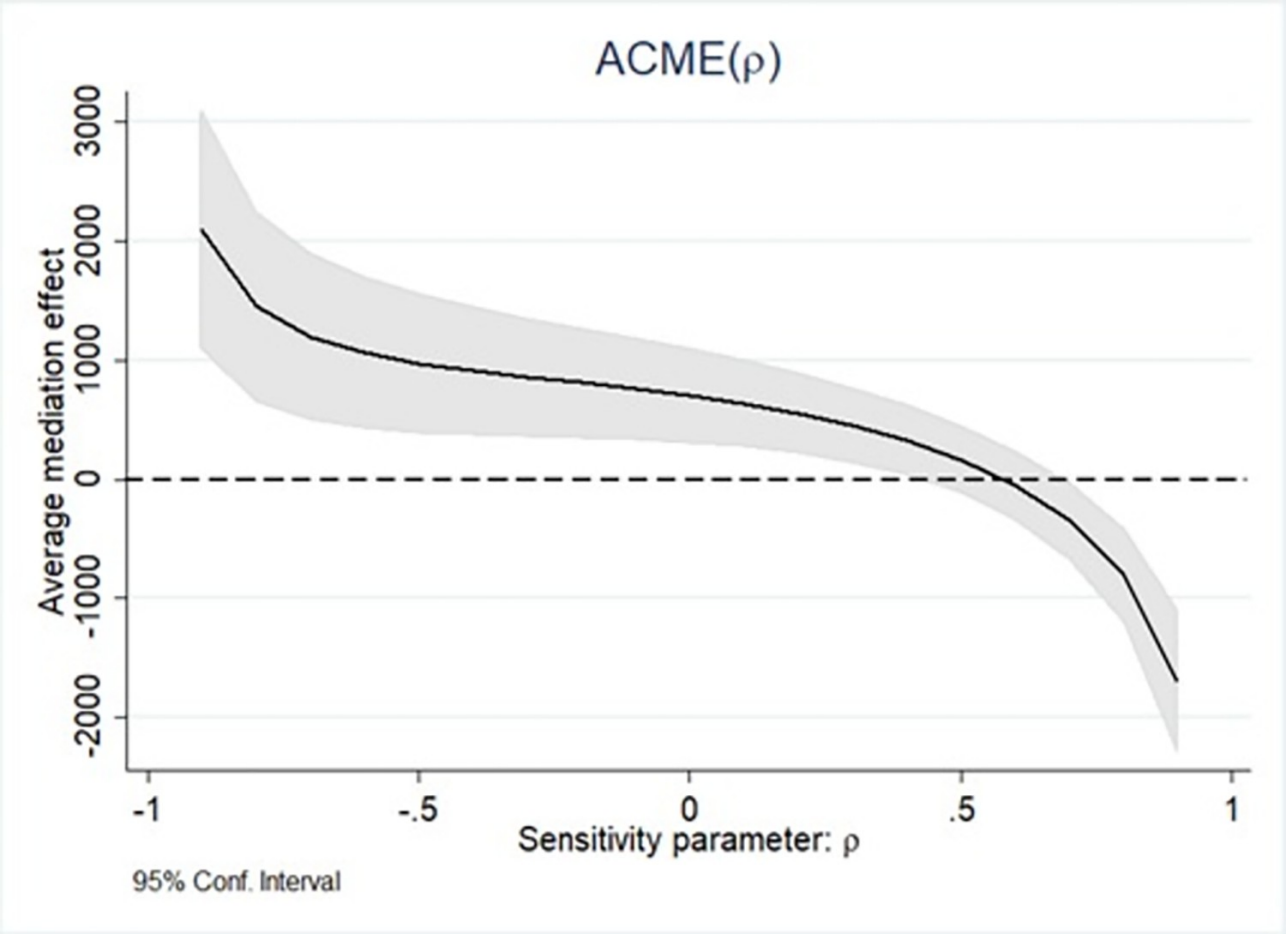
Visual presentation of sensitivity analysis for causal mediation analysis.

Besides, sensitivity analysis indicates how an estimated quantity that violates the key assumption will change for different degrees [59]. A larger value of Rho corresponds to a greater difficulty in overturning the causal mediating effect. As shown in Fig 1, the value of Rho (0.45) is large enough to confirm the credibility of the causal mediating effect.

Information technology can make up for the deficiency of the low-level integration of cultural and tourism industries. It is consistent with the view that digital technologies can promote the sustainable development of cultural and tourism industries after crisis [3]. Different from the conclusion of Li *et al*. [13] that information technology played a mediating role in alleviating the impact of epidemic on cultural and tourism industries, the findings of this paper suggest that there may be a two-way causal relationship between information technology and the integration of cultural and tourism industries. On the one hand, information technology can promote the high-level integration of cultural and tourism industries and on the other hand, high-level integration stimulates the cultural and tourism industries to apply information technology in a more effective way, which in turn significantly adding value to the tourism value chain.

### Endogeneity test

The coupling degree was used as the instrumental variable because CCD is calculated based on it and the evaluation indicators of coupling degree are different from regression control variables. Model 6 in Table 4 shows the results of 2SLS. The first-stage F statistic is 118.510, which needs to be higher than 104.7 for a conventional t-test [60]. The second-stage conventional t-test of CCD is 2.23 (bigger than 1.96, corresponding to the significance level of 5%). Therefore, the 2SLS model has passed the weak instrument test and over-identification test, indicating that IV has a strong explanatory power. Moreover, the P-value and the direction of CCD to VALUE are consistent with the fixed effect, implying that the empirical results are robust.

## Conclusions and implications

The policy of cultural and tourism integration, as an important strategy in China’s 14th Five-year Plan, contributes significantly to the tourism high-quality development. The key to high-quality tourism development lies in value addition. Based on these, the paper explored how the integration of cultural and tourism industries affected the value added to tourism value chain. By building an industrial integration evaluation system based on resources integration, markets integration and supply chains integration, this paper evaluated the integration between the cultural and tourism industries of 13 cities in Jiangsu, and applied four econometric models to analyze the impact mechanisms. The following conclusions are drawn from the findings.

Firstly, the integration of cultural and tourism industries in Jiangsu Province is generally at an imbalanced stage, with significant differences between the south and the north. Only three cities, namely Nanjing, Suzhou and Wuxi, have achieved high-level integration, all located in economically developed areas. It means that that not all cities are suitable for the policy of cultural and tourism integration, because the integration of cultural and tourism industries is ineffective in regions where the cultural industry is substantially less developed than the tourism industry.

Secondly, the integration between cultural and tourism industries not only improves tourism value chain directly but also promotes its value added indirectly through information technology. Information technology plays a mediating role in industrial integration since it can break down barriers across industries. Tourism agglomeration can intensify the promoting effect of cultural and tourism integration on the value added to the tourism value chain, and plays a positive moderating role due to the scaling effect. Thereby, information technology and tourism agglomeration can be used to adjust places that lack integration prerequisites.

Thirdly, high-level integration provides a stronger value-added effect than low-level integration. The tourism value chain may be inhibited when the development of the cultural industry lags behind that of the tourism industry by a significant gap. It seems obvious that the two industries cannot achieve high-level integration and maximize the benefits of industrial integration without engaging in synergistic development.

### Theory implications

Firstly, the tourism value chain involves multi-stakeholder activities [18] and has indirect chain-reaction effects on other economic activities [22]. Therefore, it is difficult to define the division of products in the tourism industry and the value-added process of intermediate links. Based on the tourism value chain system and tourism satellite accounts [22], this paper successfully quantified the value added to the tourism value chain by industrial integration, which is equal to the total output of the tourism subsectors minus the total tourist consumption. This finding may contribute to the quantitative research of the tourism value chain.

Secondly, Porter’s value chain model defines the actions of a firm that create value addition, while tourism is a demand-oriented business that caters to the needs of tourists. The value added to the tourism value chain can occur in four stages [61]. This paper revealed that the key to the value added to the tourism value chain is to enhance the tourist experience value, which is consistent with the idea of value shop [18]. In response to Hjalager’s horizontal coordination method based on the destination logic [16], this paper addressed the research gap between industrial integration and the tourism value chain by clarifying the relationship between the two.

Thirdly, this paper extended the application of the threshold model to different stages of industrial integration, which are usually analyzed by the average level of CCD. In general, the threshold model is used to address the link between exposure and reaction, such as the relationship between financial constraints and company investment decisions [62]. This model can automatically calculate the turning points in various stages and display the effects on the periods that follow. Therefore, it is suggested that the threshold model can be used to analyze the problems related to industrial integration in different stages.

### Policy implications

According to the impact mechanism between the cultural and tourism integration and the value added to tourism value chain, information technology and tourism agglomeration are two key driving forces for high-level integration. This theoretically supports the government to implement incentives for the integration between cultural and tourism industries. Suppose a region lacks cultural resources but has a sizable tourist base. In that case, it may benefit from the economic value generated by the integration of cultural and tourism industries through the scaling effect and external spillover effect of tourism agglomeration. For regions with abundant cultural resources but an immature tourism market, information technology can be helpful for quickly positioning and responding to the market. It can assist the actors at each node of the tourism value chain in obtaining and sharing market information, so that avoiding the crowding effect of tourism agglomeration.

Industrial integration is a stepwise process, and value-added benefits will not be achieved from initial industrial integration but require constant and comprehensive restructuring. It enlightens the government to establish a hierarchical management mechanism for the governance of both cultural and tourism industries. For regions at a low level of integration, the local government should consider implementing exit mechanisms and exploring other forms of tourism development, such as natural science tourism. For regions at a high level of integration, incentive policies can be implemented vigorously to promote the orderly and sustainable development of cultural tourism integration.

### Limitations

It should be admitted that this paper has certain limitations in terms of data sampling, model selection and value type. First, the study area is mainly concentrated in Jiangsu province, and the sample is relatively limited. Further studies are required to confirm whether the results of this paper can be generalized. Second, the possible nonlinear relationship between the integration of cultural and tourism industries and the value added to tourism value chain in different stages was ignored. In the future, the negative effect of the low-level integration of cultural and tourism industries and the measures to reduce such effect should be clarified. Lastly, this paper mainly focused on the economic value of tourism value chain, while future research can address the social or other values of tourism value chain.

## Supporting information

S1 Fig Regional differences of coupling coordination among 13 cities in Jiangsu Province. (TIF)

S2 Fig Time trends of cultural tourism integration in 13 cities in Jiangsu Province. (TIF)

S1 Table Raw data of evaluation index system for the cultural industry and tourism industry. (XLSX)

S2 Table Raw data of regression analysis of cultural tourism integration on tourism value chain. (XLSX)

## References

[1] Loulanski T, Loulanski V. The sustainable integration of cultural heritage and tourism: a meta-study. J Sustain Tour. 2011 Sep;19(7):837–62.

[2] Richards G. Creativity and tourism: The State of the Art. Ann Tour Res. 2011 Oct;38(4):1225–53.

[3] Zhao X, Xie C, Huang L, Wang Y, Han T. How digitalization promotes the sustainable integration of culture and tourism for economic recovery. Econ Anal Policy. 2023 Mar 1;77:988–1000.

[4] Clay PM, Feeney R. Analyzing agribusiness value chains: a literature review. Int Food Agribus Manag Rev. 2019 Jan 28;22(1):31–46.

[5] Adiyia B, Stoffelen A, Jennes B, Vanneste D, Ahebwa WM. Analysing governance in tourism value chains to reshape the tourist bubble in developing countries: the case of cultural tourism in Uganda. J Ecotourism. 2015 Sep 2;14(2–3):113–29.

[6] Tejada P, Santos FJ, Guzmán J. Applicability of global value chains analysis to tourism: issues of governance and upgrading. Serv Ind J. 2011 Aug;31(10):1627–43.

[7] McKercher B. Cultural tourism market: a perspective paper. Tour Rev. 2020;75(1):126–9.

[8] Scarlett HG. Tourism recovery and the economic impact: A panel assessment. Res Glob. 2021 Dec;3:100044.

[9] Fan T, Xue D. Spatial correlation of cultural industry and tourism industry in Shaanxi Province, China: LISA analysis based on coordination model. Asia Pac J Tour Res. 2020 Sep 1;25(9):967–80.

[10] Chang W. An Integrated Development Model of Cultural and Creative Industry and Rural Tourism Industry Based on Data Mining. Wirel Commun Mob Comput. 2022 Apr 25;2022:2351906.

[11] Chen H, Chen T, Li L, Chen X, Huang J. Testing Convergence of Tourism Development and Exploring Its Influencing Factors: Empirical Evidence from the Greater Bay Area in China. Sustainability. 2022 May 28;14(11):6616.

[12] Zhou Z, Yang Q, Kim DJ. An Empirical Study on Coupling Coordination between the Cultural Industry and Tourism Industry in Ethnic Minority Areas. J Open Innov Technol Mark Complex. 2020 Aug 20;6(3):65.

[13] Li X, Liang X, Yu T, Ruan S, Fan R. Research on the Integration of Cultural Tourism Industry Driven by Digital Economy in the Context of COVID-19—Based on the Data of 31 Chinese Provinces. Front Public Health. 2022 Mar 9;10:780476. doi: 10.3389/fpubh.2022.780476 35356017 PMC8959376

[14] Zhou C, Sotiriadis M. Exploring and Evaluating the Impact of ICTs on Culture and Tourism Industries’ Convergence: Evidence from China. Sustainability. 2021 Oct 25;13(21):11769.

[15] Su Z, Aaron JR, McDowell WC, Lu DD. Sustainable Synergies between the Cultural and Tourism Industries: An Efficiency Evaluation Perspective. Sustainability. 2019 Dec;11(23):6607.

[16] Hjalager AM, Tervo-Kankare K, Tuohino A. Tourism value chains revisited and applied to rural well-being tourism. Tour Plan Dev. 2016 Oct;13(4):379–95.

[17] Song H, Liu J, Chen G. Tourism Value Chain Governance: Review and Prospects. J Travel Res. 2013 Jan;52(1):15–28.

[18] Bakucz M. Tourism Value Chain Management as a Tool for Effective Tourism Destination Development The Case of Pécs ECoC 2010. Acta Univ Danub Oeconomica. 2011;7(3):46–63.

[19] Spencer JP, Safari E, Dakora EAN. An evaluation of the tourism value-chain as an alternative to socio-economic development in Rwanda, Africa. Afr J Phys Health Educ Recreat Dance. 2014;20(2:1):569–83.

[20] Zheng L, Wang H, Li G, Guo Y. Construction Scenario for a Rural Tourism Value Chain: A Case Study from Rural China. Am J Ind Bus Manag. 2021;11(1):1–18.

[21] Pesce D, Neirotti P, Paolucci E. When culture meets digital platforms: value creation and stakeholders’ alignment in big data use. Curr Issues Tour. 2019 Sep 14;22(15):1883–903.

[22] Mitchell J. Value chain approaches to assessing the impact of tourism on low-income households in developing countries. J Sustain Tour. 2012;20(3):457–75.

[23] Rylance A, Spenceley A. Reducing economic leakages from tourism: A value chain assessment of the tourism industry in Kasane, Botswana. Dev South Afr. 2017 May 4;34(3):295–313.

[24] Zhu Y. An Economic Model for Studying the Role of Cultural Industries on Social Development in Cross-border Contexts. Emerg Mark Finance Trade. 2020 May 27;56(7):1581–600.

[25] Zhou WZ, Xu YY, Zhou ZC. Management systems of flower-themed tourism in China: a value chain analysis. Axelson LE, Fernqvist F, editors. Xviii Int Symp Hortic Econ Manag. 2016;1132:113–20.

[26] Wang G, Ye L. Spatial-Temporal Pattern of Mismatch Degree of High-Quality Tourism Development and Its Formation Mechanism in Taihu Lake Basin, China. Sustainability. 2022 Apr;14(8):4812.

[27] Shi Z, Xu D, Xu L. Spatiotemporal characteristics and impact mechanism of high-quality development of cultural tourism in the Yangtze River Delta urban agglomeration. Plos One. 2021 Jun 22;16(6):e0252842. doi: 10.1371/journal.pone.0252842 34157034 PMC8219149

[28] Hu Y. The Integration and Development path of Culture and Tourism Industry based on Digital Technology Enablement. J Commer Econ. 2022;(1):182–4.

[29] Li Z, Liu H. How tourism industry agglomeration improves tourism economic efficiency? Tour Econ. 2021 May 10;135481662110091.

[30] Adachi Y. Applicability of agglomeration to tourism economics. Jpn World Econ. 2018 Sep;47:58–67.

[31] Yang Y. Agglomeration density and tourism development in China: An empirical research based on dynamic panel data model. Tour Manag. 2012 Dec;33(6):1347–59.

[32] Xiang Z. From digitization to the age of acceleration: On information technology and tourism. Tour Manag Perspect. 2018 Jan;25:147–50.

[33] Tajzadeh-Namin A. A review on value creation in tourism industry. Manag Sci Lett. 2012 Jan 1;2(1):203–12.

[34] Law R, Buhalis D, Cobanoglu C. Progress on information and communication technologies in hospitality and tourism. Int J Contemp Hosp Manag. 2014;26(5):727–50.

[35] Pietrzak M, Chlebicka A, Kraciński P, Malak-Rawlikowska A. Information Asymmetry as a Barrier in Upgrading the Position of Local Producers in the Global Value Chain—Evidence from the Apple Sector in Poland. Sustainability. 2020 Sep 23;12(19):7857.

[36] Geng Y, Wei Z, Zhang H, Maimaituerxun M. Analysis and Prediction of the Coupling Coordination Relationship between Tourism and Air Environment: Yangtze River Economic Zone in China as Example. Discrete Dyn Nat Soc. 2020 Jan 31;2020:1–15.

[37] Wang S jia, Kong W, Ren L, Zhi D dan, Dai B ting. Research on misuses and modification of coupling coordination degree model in China. J Nat Resour. 2021;36(3):793.

[38] Shen L, Huang Y, Huang Z, Lou Y, Ye G, Wong SW. Improved coupling analysis on the coordination between socio-economy and carbon emission. Ecol Indic. 2018 Nov;94:357–66.

[39] Li J, Xia J, Zuo Y, Cui J, Qiu Q, Liu X, et al. Spatiotemporal Evolution Patterns and Driving Factors of Synergistic Development of Culture, Sports, and Tourism Industries: The Case Study of China. Math Probl Eng. 2021 Nov 26;2021:1–13.

[40] Wang Q. Fixed-effect panel threshold model using Stata. Stata J Promot Commun Stat Stata. 2015;15(1):121–34.

[41] Wu Q, Zhang C, Wang H, Hao J. Study on the relationship between agglomeration of service industry and economic growth in Yangtze River Delta based on spatial econometric models. J Phys Conf Ser. 2019 Oct 1;1324(1):012089.

[42] Wang J, Ma J. Has Tourism Industry Agglomeration Improved the Total Factor Productivity of Chinese Urban Agglomerations?—The Moderating Effect of Public Epidemic. Front Public Health. 2022 Mar 17;10:854681. doi: 10.3389/fpubh.2022.854681 35372188 PMC8968943

[43] Tang R, Xiu P. The modern service industry agglomeration and tourism efficiency in China: regional difference and influencing mechanism. J Asia Pac Econ. 2021 Jul 27;1–20.

[44] Li Y, Li R, Ruan W, Liu CH. Research of the Effect of Tourism Economic Contact on the Efficiency of the Tourism Industry. Sustainability. 2020 Jul 14;12(14):5652.

[45] Feng J, Wang C, Zhao Y. Measurement and causes of cultural industry agglomeration in Jiangsu Province. AIP Conf Proc. 2023 May 5;2685(1):040021.

[46] Shen W, Huang Z, Yin S, Hsu WL. Temporal and Spatial Coupling Characteristics of Tourism and Urbanization with Mechanism of High-Quality Development in the Yangtze River Delta Urban Agglomeration, China. Appl Sci-Basel. 2022 Apr;12(7):3403.

[47] Kadarusman Y, Nadvi K. Competitiveness and Technological Upgrading in Global Value Chains: Evidence from the Indonesian Electronics and Garment Sectors. Eur Plan Stud. 2013 Jul;21(7):1007–28.

[48] Wu Z, Lai IKW, Tang H. Evaluating the Sustainability Issues in Tourism Development: An Adverse-Impact and Serious-Level Analysis. Sage Open. 2021 Oct;11(4):21582440211050384.

[49] Qu C, Timothy DJ, Zhang C. Does tourism erode or prosper culture? Evidence from the Tibetan ethnic area of Sichuan Province, China. J Tour Cult Change. 2019 Jul 4;17(4):526–43.

[50] Intason M, Lee C, Coetzee W. Examining the interplay between a hallmark cultural event, tourism, and commercial activities: A case study of the Songkran Festival. J Hosp Tour Manag. 2021 Dec;49:508–18.

[51] Zhang SN, Ruan WQ, Li YQ, Huang H. Local cultural distortion risk at tourist destinations: connotation deconstruction and theoretical construction. Curr Issues Tour [Internet]. 2023 [cited 2023 Apr 30]; Available from: https://www.webofscience.com/wos/woscc/full-record/WOS:000939508000001

[52] Dong F, Zhang S, Zhu J, Sun J. The Impact of the Integrated Development of AI and Energy Industry on Regional Energy Industry: A Case of China. Int J Environ Res Public Health. 2021 Aug 25;18(17):8946. doi: 10.3390/ijerph18178946 34501536 PMC8431408

[53] Środa-Murawska S, Grzelak-Kostulska E, Biegańska J, Dąbrowski LS. Culture and Sustainable Tourism: Does the Pair Pay in Medium-Sized Cities? Sustainability. 2021 Aug 13;13(16):9072.

[54] Yan B, Dong Q, Li Q, Yang L, Amin FU I. A study on the interaction between logistics industry and manufacturing industry from the perspective of integration field. Plos One. 2022 Mar 3;17(3):e0264585. doi: 10.1371/journal.pone.0264585 35239696 PMC8893707

[55] Han C, Hua D, Li J. A View of Industrial Agglomeration, Air Pollution and Economic Sustainability from Spatial Econometric Analysis of 273 Cities in China. Sustainability. 2023 Jan;15(9):7091.

[56] Zhang H, Zhang J, Song J. Analysis of the threshold effect of agricultural industrial agglomeration and industrial structure upgrading on sustainable agricultural development in China. J Clean Prod. 2022;341:130818.

[57] Majewska J. Inter-regional agglomeration effects in tourism in Poland. Tour Geogr. 2015 May 27;17(3):408–36.

[58] Imai K, Keele L, Tingley D. A general approach to causal mediation analysis. Psychol Methods. 2010;15(4):309–34. doi: 10.1037/a0020761 20954780

[59] Hicks R, Tingley D. Causal mediation analysis. Stata J Promot Commun Stat Stata. 2011;11(4):605–19.

[60] Lal A, Lockhart MW, Xu Y, Zu Z. How Much Should We Trust Instrumental Variable Estimates in Political Science? Practical Advice based on Over 60 Replicated Studies. SSRN Electron J. 2023;(2303):11399.

[61] Yilmaz Y, Bititci U. Performance measurement in the value chain: manufacturing v. tourism. Int J Product Perform Manag. 2006 Jul 1;55(5):371–89.

[62] Hansen BE. Threshold effects in non-dynamic panels: Estimation, testing, and inference. J Econom. 1999 Dec 1;93(2):345–68.

