# Supplementary figures and images for: How does the integration of cultural and tourism industries impact the value added to tourism value chain: Evidences from Jiangsu Province of China
Source: PLoS One. 2023 Jun 29;18(6):e0287610. doi: 10.1371/journal.pone.0287610 (PMC10309601; doi:10.1371/journal.pone.0287610)

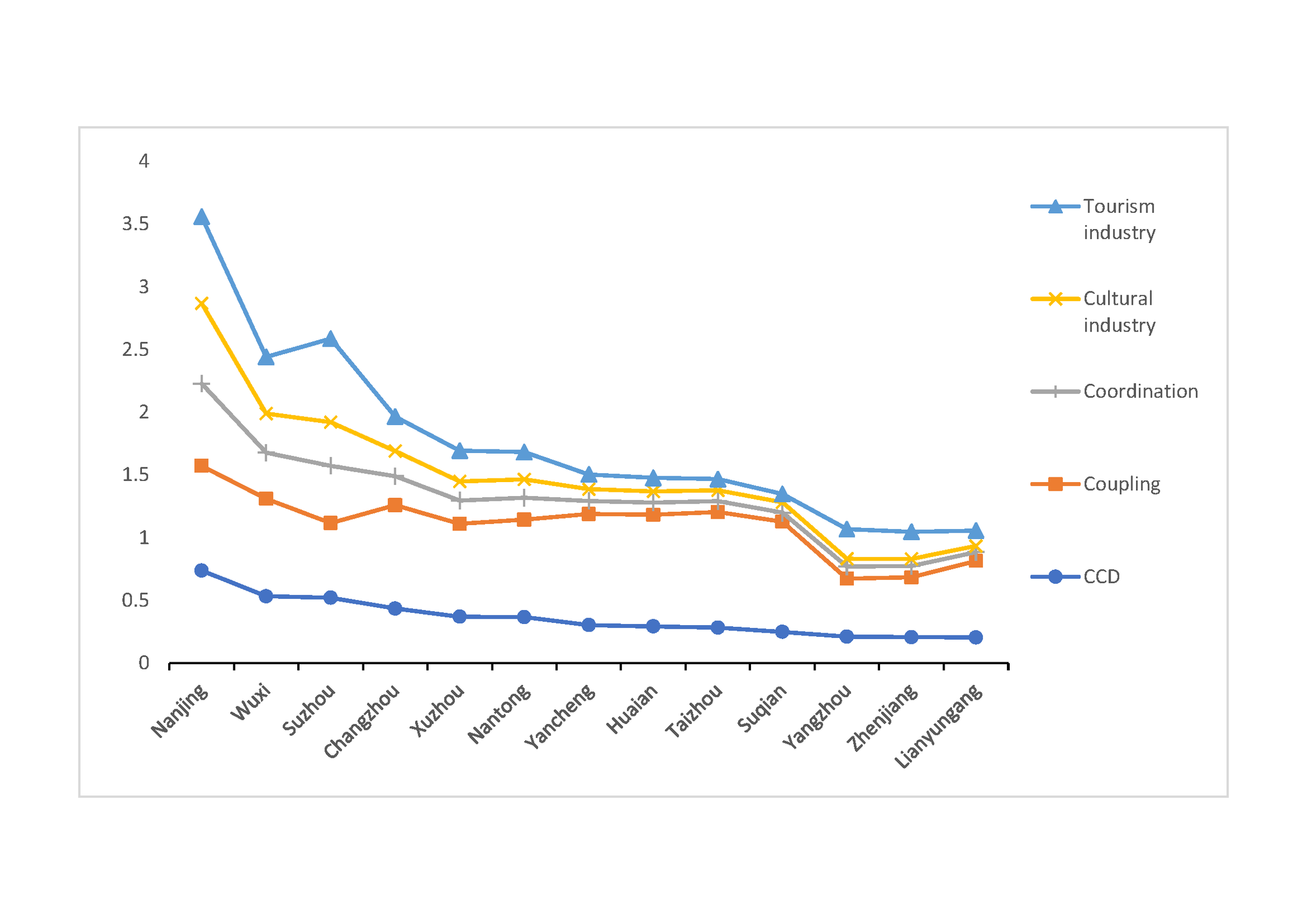

Supplement: S1 Fig — (TIF) [file pone.0287610.s001.tif]

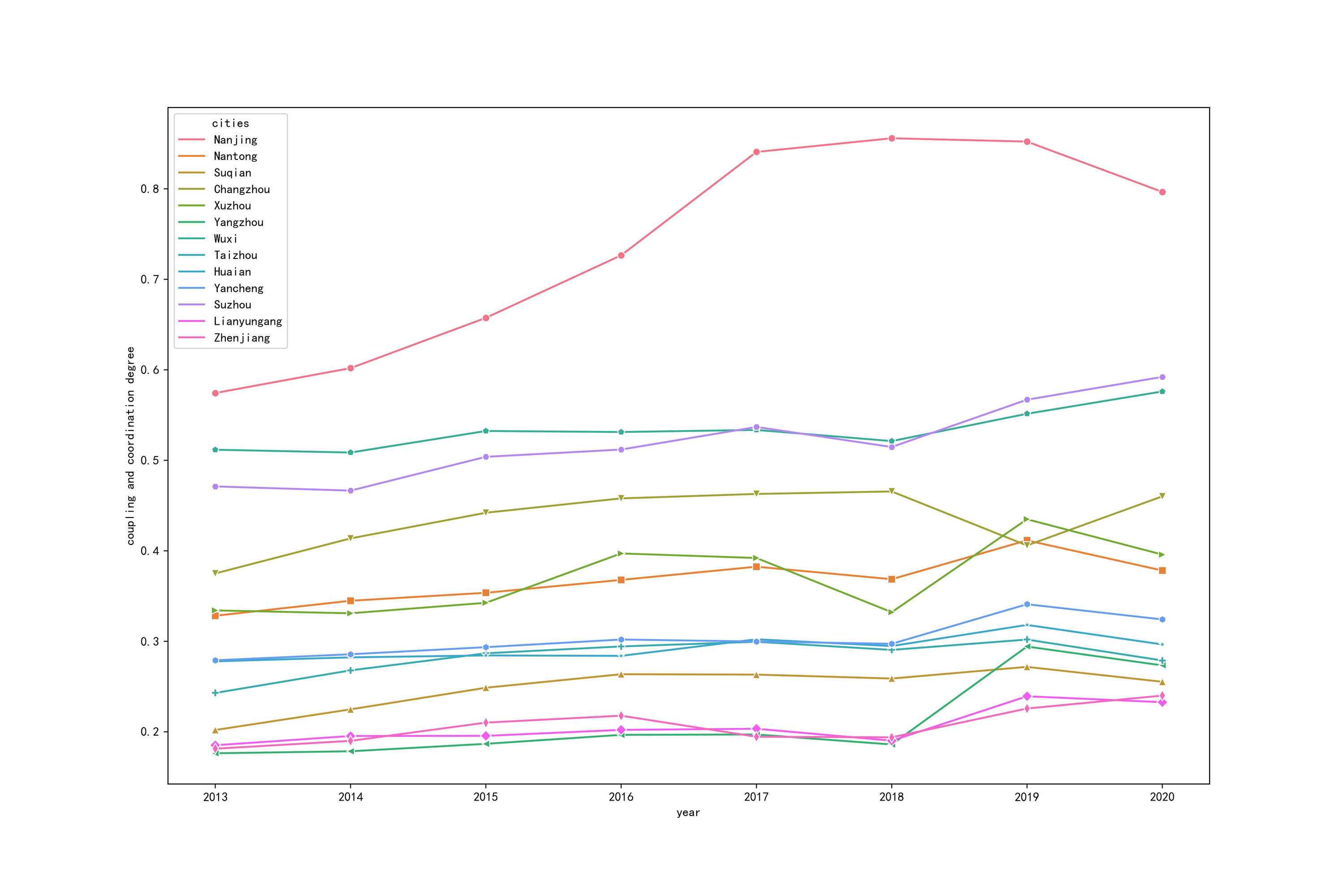

Supplement: S2 Fig — (TIF) [file pone.0287610.s002.tif]
